# Inverted polymer fullerene solar cells exceeding 10% efficiency with poly(2-ethyl-2-oxazoline) nanodots on electron-collecting buffer layers

**DOI:** 10.1038/ncomms9929

**Published:** 2015-12-14

**Authors:** Sungho Nam, Jooyeok Seo, Sungho Woo, Wook Hyun Kim, Hwajeong Kim, Donal D. C. Bradley, Youngkyoo Kim

**Affiliations:** 1 Organic Nanoelectronics Laboratory, School of Applied Chemical Engineering, Kyungpook National University, Sankyuk-dong University Road 80, Daegu 702-701, Republic of Korea; 2 Blackett Laboratory, Department of Physics and Center for Plastic Electronics, Imperial College London, London SW7 2AZ, UK; 3 Green Energy Research Division, Daegu Gyeongbuk Institute of Science and Technology, Daegu 711-873, Republic of Korea; 4 Research Institute of Advanced Energy Technology, Kyungpook National University, Daegu 702-701, Republic of Korea; 5 Departments of Engineering Science and Physics, Division of Mathematical, Physical and Life Sciences, University of Oxford, Oxford OX1 3PD, UK

## Abstract

Polymer solar cells have been spotlighted due to their potential for low-cost manufacturing but their efficiency is still less than required for commercial application as lightweight/flexible modules. Forming a dipole layer at the electron-collecting interface has been suggested as one of the more attractive approaches for efficiency enhancement. However, only a few dipole layer material types have been reported so far, including only one non-ionic (charge neutral) polymer. Here we show that a further neutral polymer, namely poly(2-ethyl-2-oxazoline) (PEOz) can be successfully used as a dipole layer. Inclusion of a PEOz layer, in particular with a nanodot morphology, increases the effective work function at the electron-collecting interface within inverted solar cells and thermal annealing of PEOz layer leads to a state-of-the-art 10.74% efficiency for single-stack bulk heterojunction blend structures comprising poly[4,8-bis(5-(2-ethylhexyl)thiophen-2-yl)benzo[1,2-b:4,5-b′]dithiophene-alt-3-fluorothieno[3,4-b]thiophene-2-carboxylate] as donor and [6,6]-phenyl-C_71_-butyric acid methyl ester as acceptor.

## Abstract (web summary)

Forming a dipole layer at the electron-collecting interface is a proposed method of efficiency enhancement in solar cells. Here, Nam *et al.* report the use of PEOz as a dipole layer, which increases the work function at the electron-collecting interface within the polymer solar cell leading to an efficiency of 10.7%.

## Graphical abstract

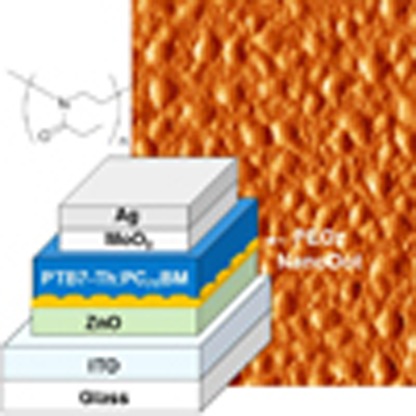

Polymer solar cells have been extensively studied because of their potential to enable high-throughput, low-temperature roll-to-roll fabrication of flexible solar modules^1^,^2^,^3^,^4^,^5^,^6^,^7^. Since the landmark early works on the concept of polymer:fullerene bulk heterojunction (BHJ) solar cells^8^,^9^,^10^,^11^,^12^,^13^,^14^, the power conversion efficiency (PCE) has been remarkably improved up to ∼10% for polymer:fullerene solar cells by introducing new light-absorbing conjugated polymers and fullerene derivatives^15^,^16^,^17^,^18^,^19^,^20^,^21^,^22^,^23^,^24^,^25^,^26^. Most of the high-efficiency polymer:fullerene solar cells adopt an inverted-type device structure, because a stable charge-collecting buffer layer can be made using metal oxides such as zinc oxide (ZnO) and titanium oxide instead of poly(3,4-ethylenedioxythiophene):poly(styrenesulfonate) (PEDOT:PSS). PEDOT:PSS is strongly acidic, leading to deterioration of the active layers and/or bottom electrodes in the case of normal-type device structure^27^,^28^. Moves have also been made to replace PEDOT:PSS with alternative hole-collecting buffer layers, such as CuSCN^29^,^30^.

In the case of inverted polymer:fullerene solar cells, further efficiency enhancement can be achieved by applying an organic interfacial layer (interlayer) between the electron-collecting buffer layer (ECBL)—typically a metal oxide—and the BHJ photogeneration layer. The dipolar nature of the interlayer results in a larger built-in potential and enhanced electron transfer from the fullerene lowest unoccupied molecular orbital^31^,^32^. Another role that such interlayers can perform is to improve adhesion between the metal oxide and BHJ layers^33^,^34^. Polymer interlayers that lead to high efficiencies can be divided into two categories. First, charged polymers with counter-ions such as PFNBr, PFN-OH, WPF-oxy-F, PFNSO, PF6NO, P3ImHT, PTMAHT and so on^35^,^36^,^37^,^38^,^39^,^40^,^41^,^42^,^43^. Concerns exist, however, over cost, tendency to aggregate and the exciton-quenching and/or charge-carrier trapping effects of the ionic species. Second, neutral polymers, for which the key example reported to date is poly(ethylene imine) (PEI) (also known as poly(ethylene imine)-ethoxylated)^44^,^45^,^46^,^47^. PEI is expected to be relatively free from exciton-quenching and/or charge-trapping effects. Interestingly, PEI is typically synthesized from poly(2-ethyl-2-oxazoline) (PEOz, Fig. 1a) by hydrolysis and/or other reactions^48^,^49^,^50^, offering the intriguing prospect of using PEOz in its place. Considering the presence of nitrogen atoms in the well-defined backbone of PEOz, it is expected that PEOz chains can act as an electron donor in a similar manner to PEI when they contact the surface of electron-accepting materials. However, PEOz has never been tried for solar cell applications, even though it has been used for biomedical applications such as drug delivery and biosensors because of its excellent biocompatibility and stealth behaviour^51^,^52^,^53^,^54^,^55^.

In this study, we report the use of PEOz as a cathode interlayer in inverted BHJ solar cells with ZnO as ECBL. The polymer:fullerene BHJ layer that we used comprises poly[4,8-bis(5-(2-ethylhexyl)thiophen-2-yl)benzo[1,2-b:4,5-b′]dithiophene-alt-3-fluorothieno[3,4-b]thiophene-2-carboxylate] (PTB7-Th) and [6,6]-phenyl-C_71_-butyric acid methyl ester (PC_71_BM). PEOz layers were spin coated from methanol and the influence of solution concentration (thickness) on film morphology and work function was investigated by atomic force microscopy (AFM), Auger electron microscopy (AEM), scanning electron microscopy (SEM), ultraviolet photoelectron spectroscopy (UPS) and electric force microscopy (EFM). A distinctive nanodot (ND) morphology was obtained for ∼4 mg ml^−1^ PEOz solution concentrations, resulting in maximum PCE=10.74%. Korea Institute of Energy Research-certified performance data for a slightly less optimized (PCE=9.6%) PEOz interlayer solar cell are presented.

## Results and Discussion

### PEOz-induced changes in ZnO work function

**Figure 1:**
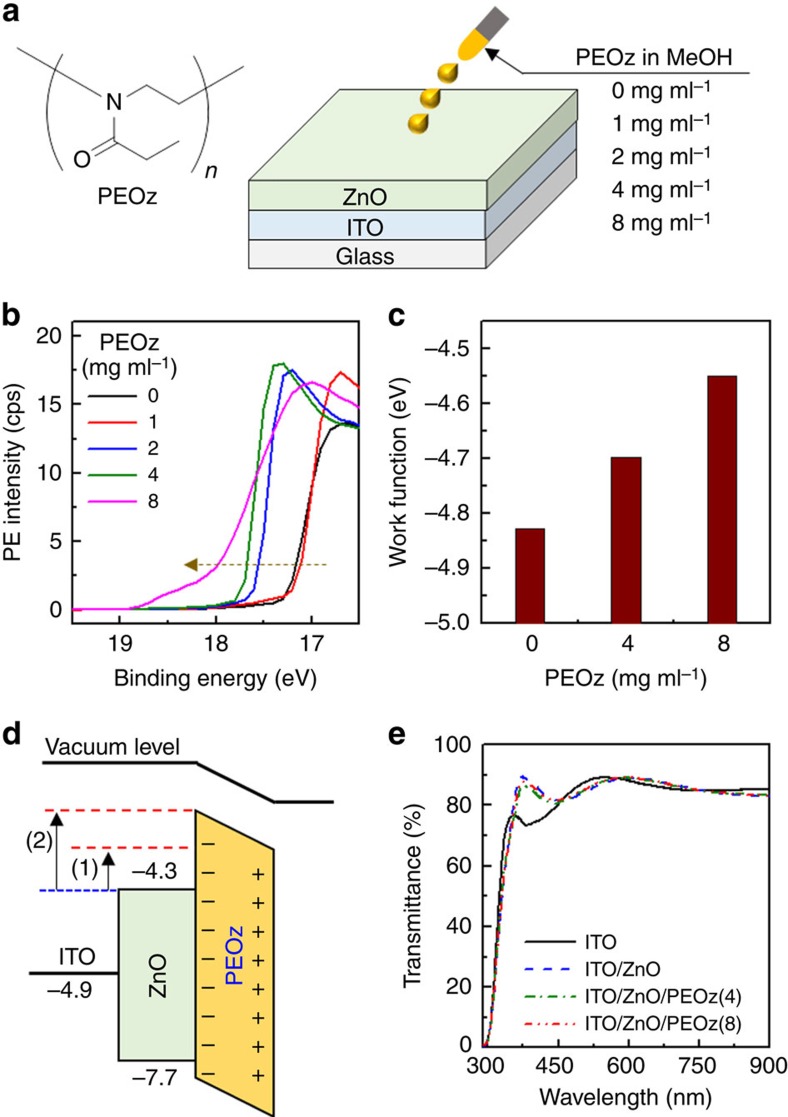
PEOz-modified ZnO layer. (**a**) Chemical structure of PEOz and schematic of PEOz coating on the ZnO ECBL. (**b**) Energy cutoff region UPS spectra as a function of PEOz concentration. (**c**) Work function energies for PEOz-coated ZnO, measured using the EFM system. (**d**) Simplified energy band diagram for the work function shift in the ZnO layer resulting from PEOz addition: (1) 0.15 eV for 4 mg ml^−1^ and (2) 0.28 eV for 8 mg ml^−1^ PEOz methanol solutions. (**e**) Optical transmittance spectra for ITO, ITO/ZnO and ITO/ZnO/PEOz, where PEOz(4) and PEOz(8) denote films deposited from 4 and 8 mg ml^−1^ solutions.

As a first step, we examined the effect of PEOz interlayers on the ECBL work function. PEOz films were spin coated (1,500 r.p.m.) from different methanol solution concentrations (Fig. 1a) and after drying the PEOz-coated ZnO samples were subjected to UPS and EFM measurements (*c.f.* details in Methods section). As observed in Fig. 1b, the cutoff part of the UPS spectrum shifted towards higher binding energy as the PEOz solution concentration increased, indicating a reduction in the magnitude of the ZnO work function with increasing coverage (film thickness)^31^,^56^,^57^. This trend was also confirmed by the EFM measurements (see Fig. 1c), which are performed in an inert (nitrogen) environment at atmospheric pressure so that actual work function change can be measured under similar conditions to those appertaining following coating of the active layer. The EFM data reveal that the ECBL work function shifts by∼0.15 eV (from −4.83 to −4.68 eV) and 0.28 eV (from −4.83 to −4.55 eV), respectively, following the spin coating of 4 and 8 mg ml^−1^ PEOz solutions. The reduction in work function associated with the PEOz dipole layer is illustrated in the simplified energy band diagram of Fig. 1d, in which the valence band (7.7 eV) and conduction band (4.3 eV) energy of the ZnO layer was obtained from the UPS spectrum in Fig. 1b and its optical band gap (see Methods section)^56^,^57^,^58^,^59^. We note that the discrepancy in work function between the UPS and EFM data can be attributed primarily to the different measurement environments (*in vacuo* for the UPS measurement and in ambient for the EFM measurement) as previously well documented^58^,^59^,^60^. Figure 1e shows 300–900 nm optical transmittance spectra for ITO, ITO/ZnO and ITO/ZnO/PEOz samples, where 4 and 8 mg ml^−1^ PEOz data are labelled PEOz(4) and PEOz(8), respectively. The average transmittance between 400 and 900 nm exceeds 80% for all samples, with the optical quality evident from the Fabry–Perot interference fringes. This result confirms that the addition of a PEOz interlayer on top of the ZnO ECBL does not harm its optical transmittance, ensuring that there should be a negligible effect on the light intensity reaching the PTB7-Th:PC_71_BM BHJ layer.

### PEOz-induced changes in solar cell performance

**Figure 2:**
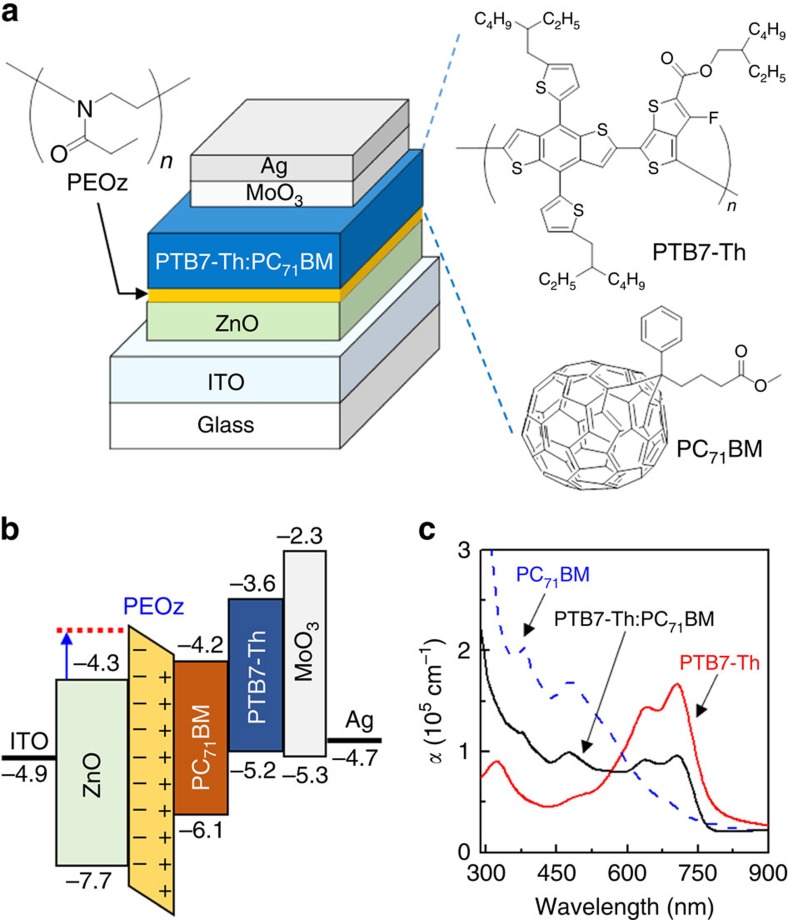
Device/energy band structure and absorption coefficient data. (**a**) Solar cell device stack and chemical structures for PTB7-Th, PC_71_BM and PEOz. (**b**) Flat energy band diagram for the PTB7-Th:PC_71_BM BHJ solar cells with ZnO/PEOz ECBL/interlayer. The conduction band and valence band energy values of the ZnO layer were measured in this work (see the Methods section). The blue arrow denotes the shift in ZnO work function caused by the PEOz. (**c**) Absorption coefficient (*α*) spectra for pristine films of PTB7-Th and PC_71_BM, and for a BHJ PTB7-Th:PC_71_BM blend film.

Based on the observed changes in ZnO work function (Fig. 1), inverted PTB7-Th:PC_71_BM BHJ solar cells were fabricated as shown in Fig. 2a. The PEOz solution concentration was again varied from 0 to 8 mg ml^−1^, to investigate the interlayer coverage (thickness) effect on solar cell performance. Taking into account the work function shift presented in Fig. 1d, the overall flat energy band diagram for our PTB7-Th:PC_71_BM solar cells can be represented as per Fig. 2b. It is useful to note that relatively small changes in the conduction band energy level (work function) of the n-type ZnO layer will significantly affect the device built-in potential (electric field). A close look at the optical absorption spectra in Fig. 2c shows that the PTB7-Th component has a vibronically structured absorption band spanning the range from ∼550 to 800 nm, coincident with the high transmittance range of the ITO/ZnO/PEOz stack.

**Figure 3:**
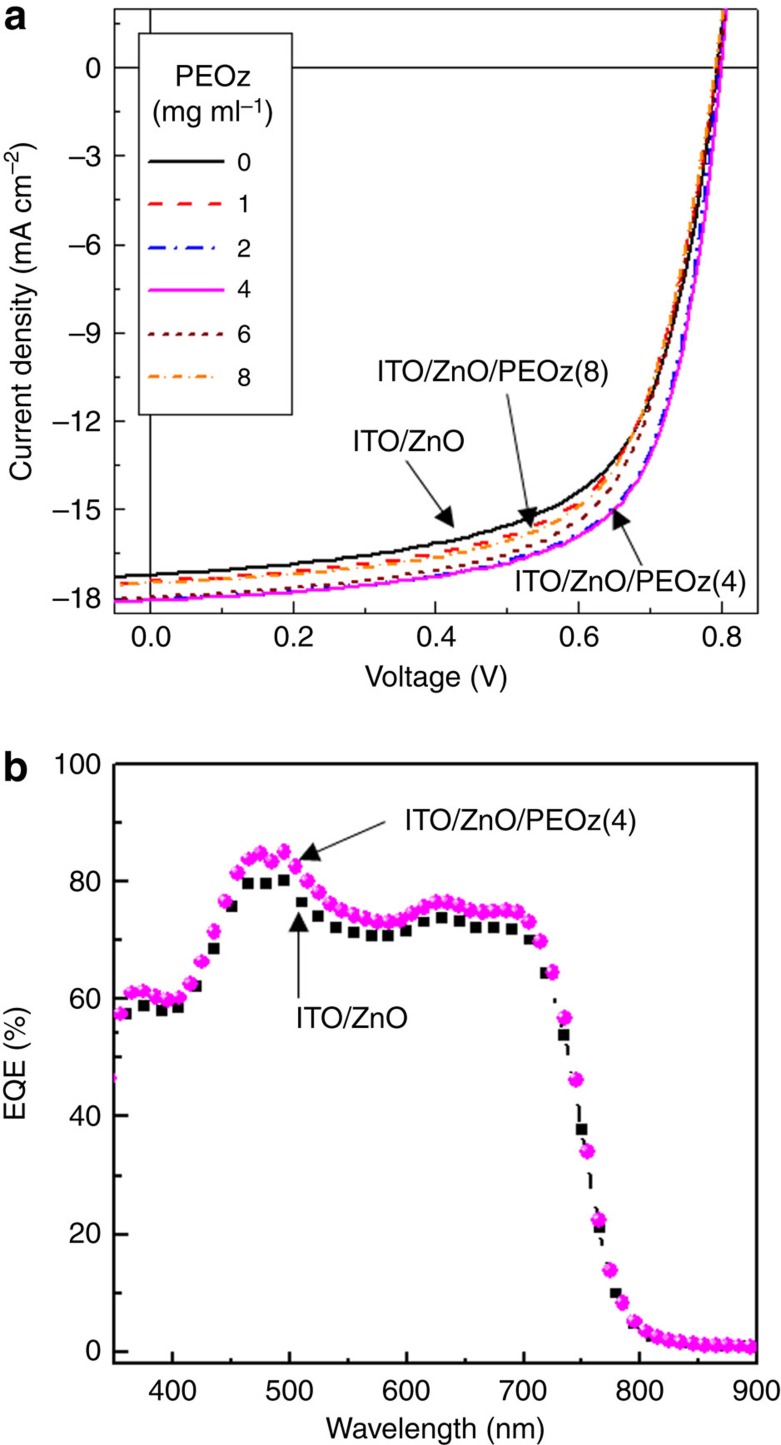
Solar cell performances. (**a**) Light (air mass 1.5G, 100 mW cm^−2^) *J*–*V* curves for PTB7-Th:PC_71_BM solar cells fabricated using PEOz solution concentrations in the range 1–8 mg ml^−1^. Data are also shown for a control device with no PEOz interlayer. (**b**) EQE spectra for PTB7-Th:PC_71_BM solar cells with a bare ZnO ECBL and with a PEOz(4)-coated ZnO ECBL.

As shown in Fig. 3a, the current density–voltage (*J*–*V*) curves under simulated solar light (air mass 1.5 G, 100 mW cm^−2^) illumination noticeably improved on addition of a PEOz layer on top of the ZnO ECBL. However, the improvement was not monotonic in PEOz concentration: the best performance was measured for the PEOz(4) device (ITO/ZnO/PEOz(4)/PTB7-Th:PC_71_BM/MoO_3_/Ag) and higher concentrations gave worse performance. Considering the work function changes for the ZnO layer in the presence of PEOz (see Fig. 1), PEOz(8) devices might have been expected to perform better than PEOz(4) devices. This result may reflect that the thicker PEOz layer could relatively restrict electron transport to the ZnO ECBL from the PTB7-Th:PC_71_BM BHJ layer, owing to a slightly higher electrical resistance. This would be consistent with the observation that the change in short circuit current density (*J*_SC_) was more pronounced than that for open circuit voltage (*V*_OC_). An enhanced *J*_SC_ for the PEOz(4) device was also confirmed by external quantum efficiency (EQE) spectra measurements (see Fig. 3b). The EQE value is higher for the PEOz(4) device than the control device (ITO/ZnO/PTB7-Th:PC_71_BM/MoO_3_/Ag) across the entire wavelength range, suggesting that the effect is not predominantly interference related. This result supports a conclusion that the improved solar cell performance is mainly attributed to enhanced charge (electron) collection in the presence of a PEOz interlayer due to the increased built-in potential represented in Fig. 1.

**Figure 4:**
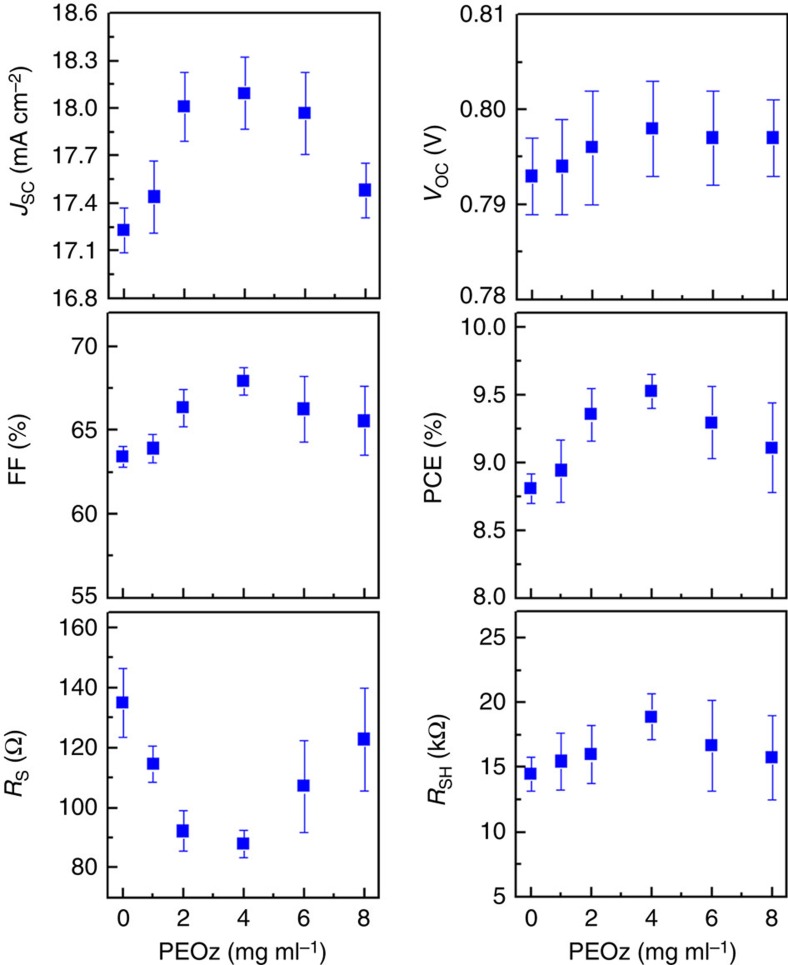
PEOz coating dependence of solar cell parameters. *J*_SC_, *V*_OC_, FF, PCE, *R*_S_ and *R*_SH_ for the PTB7-Th:PC_71_BM solar cells as a function of the PEOz concentration (N.B. these data were taken from the light *J*–*V* curves in Fig. 3 with error bars for more than 15 devices).

The detailed variation in solar cell parameter values as a function of PEOz concentration (extracted from the data shown in Fig. 3a) is shown in Fig. 4. In all cases, there is a non-monotonic variation, with 4 mg ml^−1^ providing the extreme value for each parameter. The short circuit current (*J*_*sc*_) peak is mirrored by a fill factor (FF) maximum and a series resistance (*R*_S_) minimum that are consistent with otherwise electron transport-limited behaviour. The solar cell shunt resistance (*R*_SH_) peak at 4 mg ml^−1^ PEOz concentration also signals an advantageous reduction in leakage current for this device structure.

In contrast, the *V*_OC_ value only very slightly increases up to 4 mg ml^−1^ and is then almost constant for higher concentrations; the maximum is only marginally optimal in that regard. The rising *V*_OC_ value is consistent with the work-function shifts seen in UPS measurements but the magnitude of the effect is not. *V*_OC_ increases at best by 0.005 eV compared with a work-function shift of 0.15 eV for PEOz(4) relative to PEOz(0) devices (see Fig. 1). In addition, the work-function shift continues, reaching 0.28 eV for PEOz(8), whereas *V*_OC_ is saturated beyond PEOz(4). This strongly suggests that other energy levels limit the open circuit voltage, because the offset energy between the PTB7-Th highest occupied molecular orbital and PC_71_BM lowest unoccupied molecular orbital is∼1 eV^61^,^62^, and the thicker PEOz(8) layer inevitably has a relatively higher electrical resistance.

Finally, the solar cell PCE significantly improved from 8.81 to 9.53% by depositing a PEOz(4) film (see Table 1). This 9.53% PEOz(4) device was subsequently tested and certified at 9.57% by the National Solar Cell Accreditation Center of the Korea Institute for Energy Research (see Supplementary Fig. 1). Additional experiments were undertaken to assess the ubiquity of PEOz and specifically the optimal 4 mg ml^−1^ concentration. In particular, poly[[4,8-bis[(2-ethylhexyl)oxy]benzo[1,2-b:4,5-b′]dithiophene-2,6-diyl][3-fluoro-2-[(2-ethylhexyl) carbonyl]thieno[3,4-b]thiophenediyl]] (PTB7) was substituted for the PTB7-Th donor used here and BHJ blend solar cells were fabricated, again with PC_71_BM as the acceptor. As shown in Supplementary Fig. 2, the best performance for the PTB7:PC_71_BM blend was also obtained for PEOz(4) solar cells.

### Surface morphology of the PEOz-modified ZnO ECBL

**Figure 5:**
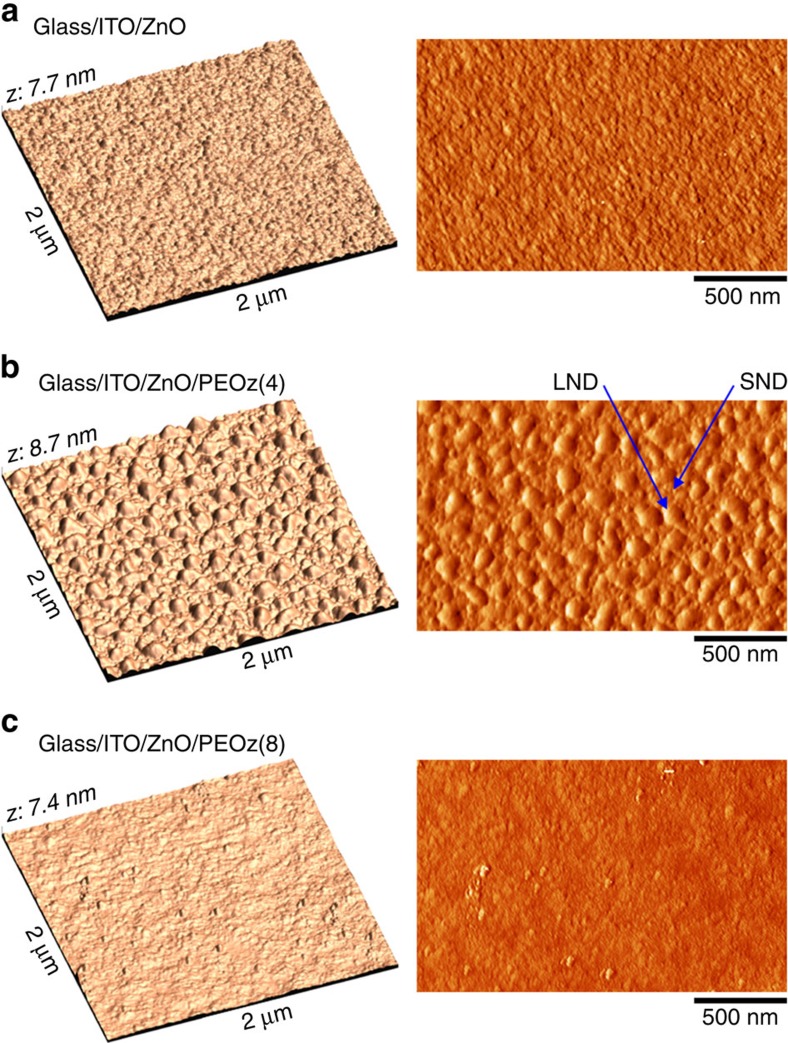
AFM measurements of the surface morphology. Three-dimensional height-mode (left) and two-dimensional phase-mode (right) AFM images. (**a**) Glass/ITO/ZnO, (**b**) glass/ITO/ZnO/PEOz(4) and (**c**) glass/ITO/ZnO/PEOz(8). ‘SND' and ‘LND' in **b** represent ‘small NDs' and ‘large NDs', respectively.

**Figure 6:**
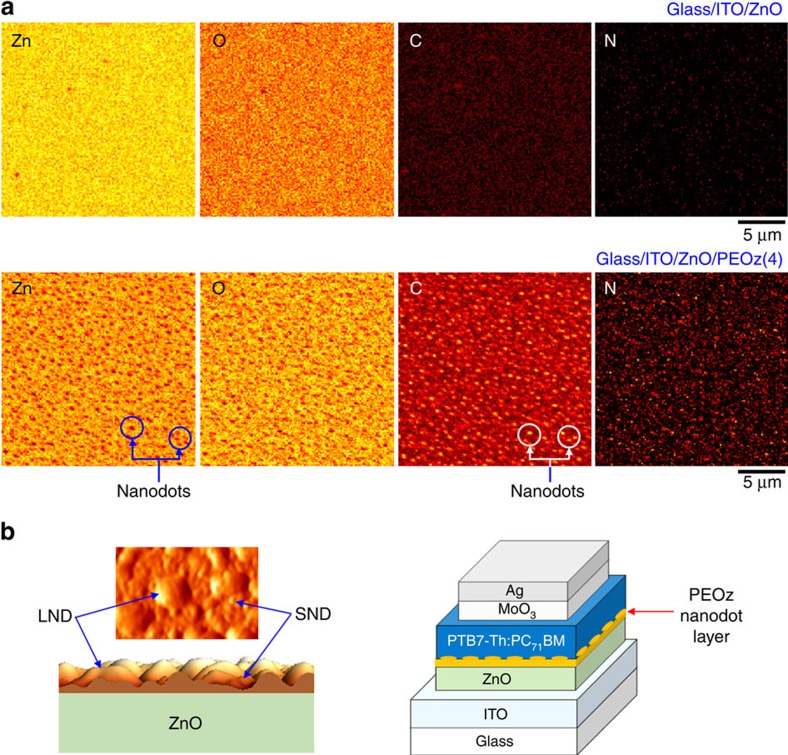
AEM measurements of the surface atom distribution and formation of NDs. (**a**) AEM images for individual atoms (zinc (Zn), oxygen (O), carbon (C) and nitrogen (N)). (**b**) Illustration for the proposed formation of the PEOz(4) nanolayer comprising SNDs and LNDs, and a contiguous coating between them.

The non-monotonic dependence of solar cell performance parameters on PEOz solution concentration suggests that more needs to be known about the microstructure and morphology of the films deposited. To investigate how such additional factors may contribute to the improved device performance, alongside a lower ZnO ECBL work function, the surface morphology of PEOz-coated ZnO was examined using AFM measurements. As shown in Fig. 5a, a fine-grain morphology was measured for bare ZnO (without the PEOz layer), with a root-mean-square roughness of 0.88 nm. A striking morphology change was found for the ZnO/PEOz(4) samples, as shown in Fig. 5b, where randomly distributed NDs cover the whole surface (see also the SEM images in Supplementary Fig. 3). In closeup, the AFM (Supplementary Fig. 4) and SEM images (Supplementary Fig. 3 right) reveal the coexistence of both small NDs (SNDs) and large NDs (LNDs) (the diameter of LNDs and SNDs is 80∼120 and 10∼20 nm, respectively). As shown in Fig. 6a, the AEM measurements confirmed the co-existence of SNDs and LNDs on the ZnO/PEOz(4) sample surface (note that ‘N' and ‘C' atoms are present only in the PEOz polymer). In addition, the AEM images suggest that the spaces between NDs may also be covered by a thin PEOz(4) nanolayer as indicated by the (colour) difference between the bare ZnO and PEOz(4)-coated ZnO surfaces. In contrast, the ZnO/PEOz(8) samples show a more uniform surface morphology that is less rough than the ZnO layer and pronouncedly different from the ZnO/PEOz(4) case. However, the presence of a pit-like defect on the PEOz(8) surface might be responsible for the relatively lower shunt resistance of devices (see Fig. 4).

The reported AFM and AEM measurements raise two questions: whether and how the ND morphology might contribute to the enhanced performance of PEOz(4) PTB7-Th:PC_71_BM solar cells. At the present time we are not able to directly answer these questions but we note that charge (electron) transport from the BHJ layer to the ZnO ECBL might, for instance, be affected by the influence of the ND structure on local electric fields. In addition (or alternatively), the physical contact (adhesion) to the BHJ blend and/or its effective active area might be enhanced by an increase in PEOz surface area. This is schematically illustrated in Fig. 6b but we note that it is difficult to probe such a situation using standard measurements. As shown in Supplementary Fig. 5 the surface nanomorphology of PTB7-Th:PC_71_BM BHJ films is little altered (other than in terms of its surface roughness) by being deposited on top of a bare ZnO ECBL or one coated with a PEOz(4) or PEOz(8) film. In addition, high-resolution transmission electron microscope images of focused ion beam prepared PEOz(4) device cross-sections only vaguely reveal the presence of the PEOz film (see Supplementary Fig. 6). Further detailed studies will be required to gain a clearer insight but our initial considerations suggest that the role of PEOz ND structures may be different from the conventional island-interface effect bestowed by metal (Ag) clusters, conical island titanium oxide (TiO_2_), and silicon (Si) nanodots, in which surface plasmon and effects have been proposed to play a significant role^63^,^64^,^65^.

### Further device optimization and stability (lifetime) studies

**Figure 7:**
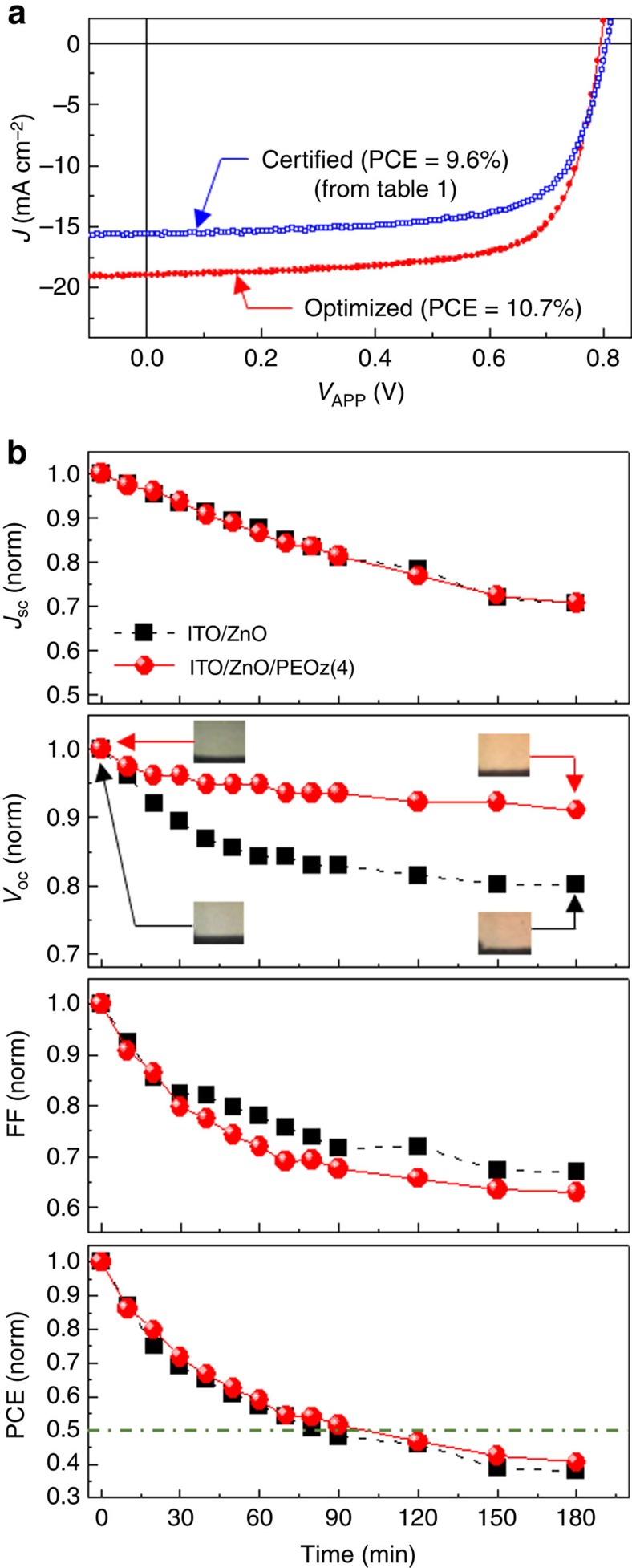
Fully optimized device performance and stability data. (**a**) Light *J*–*V* characteristics of a fully optimized PTB7-Th:PC_71_BM solar cell (*J*_SC_=19.0 mA cm^−2^, *V*_OC_=0.794 V, FF=71.2%, PCE=10.74%) together with certified data for a 9.6% PCE cell fabricated with a simple soft-baking step (without the optimized PEOz annealing step). (**b**) Change of normalized solar cell parameters for PEOz(4) and PEOz-free solar cells as a function of exposure time under continuous AM1.5G, 100 mW cm^−2^ illumination. The inset photographs in the *V*_OC_ graph show the striking colour change of the PTB7-Th:PC_71_BM BHJ that accompanies the decrease in performance.

To increase solar cell performance even more, the ZnO/PEOz(4) layer was thermally annealed before deposition of the BHJ blend film; this can lead to better adhesion between the ZnO ECBL and the PEOz interlayer. As shown in Supplementary Fig. 7, 120 °C was found to be the optimal annealing temperature and fabrication of a set of devices using this protocol yielded ∼10.7% PCE (see Fig. 7a, where *V*_OC_=0.794 V, *J*_SC_=19.0 mA cm^−2^ and FF=71.2%, and hence PCE=10.74%). PCE=10.74% represents state-of-the-art performance for a single-stack polymer:fullerene solar cell, as summarized in Supplementary Table 1. Our 10.74% solar cells were next subjected to a brief stability test under continuous illumination with simulated solar light (air mass 1.5 G, 100 mW cm^−2^). As shown in Fig. 7b, both the optimized PEOz(4) device and the control device (bare ZnO ECBL) exhibited a rapid decrease in *J*_SC_ with illumination time, whereas FF was marginally more stable for the optimized device. The *V*_OC_ trend was different in that the optimized PEOz(4) device showed a significantly slower reduction, probably reflecting the PEOz ND layer's role in retarding interfacial degradation. As a result, the overall PCE stability of the optimized PEOz(4) device (*τ*_1/2_∼98 min) was marginally better than for the control device (*τ*_1/2_∼81 min). Nevertheless, in reality both devices exhibited poor stability; this can be ascribed directly to device degradation, as it was evident that continuous illumination drastically alters the BHJ film colour (see inset photographs in Fig. 7b). In this regard, the poor stability is likely to be associated with PTB7-Th, mirroring the known instability of the closely related PTB7 polymer^66^,^67^,^68^.

## Conclusion

In summary, coating a ZnO ECBL with PEOz leads to formation of an interfacial dipole layer that induces a significant change in work function; reductions of 0.15 and 0.28 eV are achieved for PEOz(4) and (8) films, respectively. The corresponding performance of inverted ZnO-ECBL-containing PTB7-Th:PC_71_BM BHJ solar cells is noticeably improved (certified PCE ∼9.57%) by insertion of the PEOz interlayer but the improvement peaks for films prepared from 4 mg ml^−1^ solutions. Beyond this concentration there is a decline or (for *V*_oc_) saturation in cell parameters, flagging the importance of factors other than the change in work function. In addition, even for PEOz(4) films, the observed increase in *V*_OC_ is rather smaller than would have been expected from the work function shift, probably owing to the practical difference between film surface (no influence of electron transport and contact resistance) and bulk device property (strong influence of electron transport and contact resistance). The morphology/microstructure of the deposited PEOz films was, in particular, found to have a key influence on device function. A characteristic ND-like surface morphology was observed for the PEOz(4)-coated ZnO ECBL, leading to an increased contact surface area with, and an enhanced electron collection from, the BHJ blend. This ND morphology is absent for PEOz(8)-coated ZnO ECBL samples, with the corresponding absence of any increase in *V*_OC_ and *J*_SC_ then attributable to relatively higher electrical resistance. Our results have clearly demonstrated the correlation between solar cell performance and the morphology change, which can be controlled by varying the concentration of the PEOz solution. Additional optimization by thermal annealing of the PEOz(4)-coated ZnO ECBL at 120 °C resulted in PCE=10.74%, one of the best efficiencies reported for a single-stack polymer:fullerene solar cell. Annealing at higher temperatures still (150 °C, data not shown) was, however, then detrimental to device performance, resulting in a loss of the desirable ND microstructure. These results demonstrate a clear correction between our solar cell performance and the PEOz film morphology, controlled both by varying the PEOz solution concentration and by annealing. Future work will need to focus on enhancing device stability but this may require replacement of the PTB7-Th donor polymer in the BHJ blend, which, similar to the closely related PTB7 donor polymer, appears to be highly susceptible to photo-oxidation. The use of PEOz with other active solar cell materials will be the subject of ongoing studies.

## Methods

### Materials and solutions

PTB7-Th (weight-average molecular weight=126 kDa, polydispersity index=2.5) and PC_71_BM (purity>99%) were purchased from 1-Material (Canada) and Nano-C (USA), respectively. PEOz (weight-average molecular weight=50 kDa, polydispersity index=3–4) was supplied by Sigma-Aldrich (USA) and used without further purification. The PEOz solutions were prepared by varying the concentration up to 8 mg ml^−1^ in methanol. Binary polymer:fullerene solutions were prepared using chlorobenzene as a solvent in the presence of 1,8-diiodooctane (chlorobenzene:1,8-diiodooctane =97:3 by volume) at a solid concentration of 20 mg ml^−1^ (PTB7-Th:PC_71_BM=1:1.5 by weight) and were vigorously stirred at room temperature for 12 h before spin coating. The ZnO precursor solutions were prepared by dissolving zinc acetate dihydrate (Sigma-Aldrich, 1 g) and ethanolamine (Sigma-Aldrich, 0.28 g) in 2-methoxyethanol (Sigma-Aldrich, 10 ml) and stirred at 60 °C for 3 h and then at room temperature for 12 h before spin coating.

### Thin film and device fabrication

ITO-coated glass substrates (sheet resistance=10 Ω cm^−2^) were patterned by photolithography/etching processes. The pre-patterned ITO-glass substrates were cleaned using acetone and isopropyl alcohol in an ultrasonic cleaner, followed by drying under flowing nitrogen. The dried ITO-glass substrates were treated inside an ultraviolet–ozone chamber for 20 min, to remove any remnant organic residues on the surface of the substrates and to make the ITO surface hydrophilic. ZnO precursor solutions were spin coated onto the cleaned ITO-glass substrates and the resulting ITO/ZnO samples were annealed at 200 °C for 1 h in air. After cooling down to room temperature, the PEOz solutions were spin coated onto the surface of the ZnO layer, followed by soft-baking for 15 min at 60 °C (in the case of unoptimized structures) or annealing for 15 min at 120 °C (in the case of fully optimized structures) or (detrimentally) at 150 °C. The PEOz-coated samples were transferred to a nitrogen-filled glove box for the active layer coating. The PTB7-Th:PC_71_BM BHJ layers were spin-coated on top of the PEOz-coated ZnO layers and dried for 20 min inside the glove box. Next, these samples were transferred to a vacuum chamber in the argon-filled glove box, where MoO_3_ (10 nm) and Ag (80 nm) were sequentially deposited on top of the BHJ layers through a shadow mask in a vacuum of 2 × 10^−6^ Torr. The active area of devices was 0.05 or 0.09 cm^2^. For the measurement of surface morphology and nanostructure, all samples were prepared using the ITO-glass substrates in the same way as for the device fabrication, whereas the samples were prepared by spin coating on quartz substrates for optical measurements. Attempts to measure the exact thickness of the PEOz layers gave unreliable results, because the PEOz layers were too thin.

### Measurements

The thickness of films was measured using a surface profiler (Alpha Step 200, Tencor Instruments), whereas the optical absorption and transmittance of film samples were measured using an ultraviolet-visible spectrometer (Optizen 2120, MECASYS). The UPS spectra were measured using an ultra-high vacuum UPS system (ESCALAB 250Xi, Thermo Scientific) at ∼1 × 10^−9^ mbar with a He I (21.2 eV) ultraviolet source: all samples were biased at −5 V, whereas the energy scale of the UPS spectra was calibrated to the Fermi level of a thermally evaporated, cleaned Ag substrate. The valence band energy of the ZnO layer was obtained as 7.7 eV from the low binding energy part of corresponding UPS spectrum after calibration with the clean Ag reference, whereas the conduction band energy of the ZnO layer was calculated by subtracting its optical band gap (3.4 eV) (refer to Fig. 1e) from the valence band energy. The valence band maximum of the PEOz-coated ZnO layers was calculated using the equation, *E*_VBM_=*P*_IN_−(*E*_CF_−*E*_ON_), where *E*_VBM_, *P*_IN_, *E*_CF_ and *E*_ON_ are the valence band maximum, the incident photon energy (21.2 eV), the binding energy at cutoff region and the onset binding energy, respectively^69^. The work functions of the ZnO and ZnO/PEOz nanolayers were also measured using an EFM (XE-150, Park Systems) and calibrated against highly ordered pyrolytic graphite. The surface morphology was measured using an AEM (scanning Auger nanoprobe, PHI700, Physical Electronics), AFM (Nanoscope IIIa, Digital Instruments) and SEM (S-4800, Hitachi). Device cross-sections were measured using a high-resolution transmission electron microscope (Titan G2 ChemiSTEM Cs Probe, FEI Company) equipped with a focused ion beam system (Versa3D LoVac, FEI Company). The *J*–*V* curves of devices were measured using a solar cell measurement system equipped with a solar simulator (92250A-1000, Newport-Oriel) and an electrometer (Model 2400, Keithley). The EQE measurements were carried out using a specialized EQE measurement system equipped with a light source (Tungsten-Halogen lamp, 150W, ASBN-W, Spectral Products) and a monochromator (CM110, Spectral Products).

## Additional information

**How to cite this article:** Nam, S. *et al.* Inverted polymer fullerene solar cells exceeding 10% efficiency with poly(2-ethyl-2-oxazoline) nanodots on electron-collecting buffer layers. *Nat. Commun.* 6:8929 doi: 10.1038/ncomms9929 (2015).

## Supplementary Material

Supplementary Information Supplementary Figures 1-7, Supplementary Table 1 and Supplementary References.

## Figures and Tables

**Table 1:** Solar cell performances as a function of PEOz concentration in methanol.

**PEOz (mg** **ml**^−1^**)**	***V***_**OC**_ **(V)**	***J***_**SC**_ **(mA** **cm**^−2^**)**	**FF (%)**	**PCE (%)**	***R***_**S**_ **(Ω)**	***R***_**SH**_ **(kΩ)**
0	0.793 (0.004)	17.23 (0.14)	63.42 (0.64)	8.81 (0.11)	135.0 (11.5)	14.47 (1.29)
1	0.794 (0.005)	17.44 (0.23)	63.92 (0.83)	8.94 (0.23)	114.5 (6.1)	15.43 (2.20)
2	0.796 (0.006)	18.01 (0.22)	66.36 (1.11)	9.35 (0.20)	92.3 (6.8)	15.98 (2.25)
4	0.798 (0.005)	18.10 (0.23)	67.95 (0.82)	9.53 (0.12)	88.0 (4.5)	18.89 (1.78)
4*	0.806	17.21	68.97	9.57	85.3	19.2
6	0.797 (0.005)	17.97 (0.26)	66.27 (1.97)	9.29 (0.26)	107.2 (15.3)	16.68 (3.53)
8	0.797 (0.004)	17.48 (0.17)	65.56 (2.05)	9.11 (0.33)	122.8 (17.2)	15.74 (3.25)

FF, fill factor; *J*_SC_, short circuit current density; PCE, power conversion efficiency; PC_71_BM, [6,6]-phenyl-C_71_-butyric acid methyl ester; PEOz, poly(2-ethyl-2-oxazoline); PTB7-Th, poly[4,8-bis(5-(2-ethylhexyl)thiophen-2-yl)benzo[1,2-b:4,5-b′]dithiophene-alt-3-fluorothieno[3,4-b]thiophene-2-carboxylate] (PTB7-Th); *R*_S_, series resistance; *R*_SH_, cell shunt resistance; *V*_OC_, open circuit voltage; ZnO, zinc oxide.

‘4*' denotes the certified results for the inverted PTB7-Th:PC_71_BM solar cells with the ZnO/PEOz(4) layer, which were measured in the National Solar Cell Accreditation Center of Korea Institute of Energy Research.
