# Supplementary material for: Inverted polymer fullerene solar cells exceeding 10% efficiency with poly(2-ethyl-2-oxazoline) nanodots on electron-collecting buffer layers
Source: Nat Commun. 2015 Dec 14;6:8929. doi: 10.1038/ncomms9929 (PMC4682173; doi:10.1038/ncomms9929)
Supplement: Supplementary Information — Supplementary Figures 1-7, Supplementary Table 1 and Supplementary References. [file ncomms9929-s1.pdf]

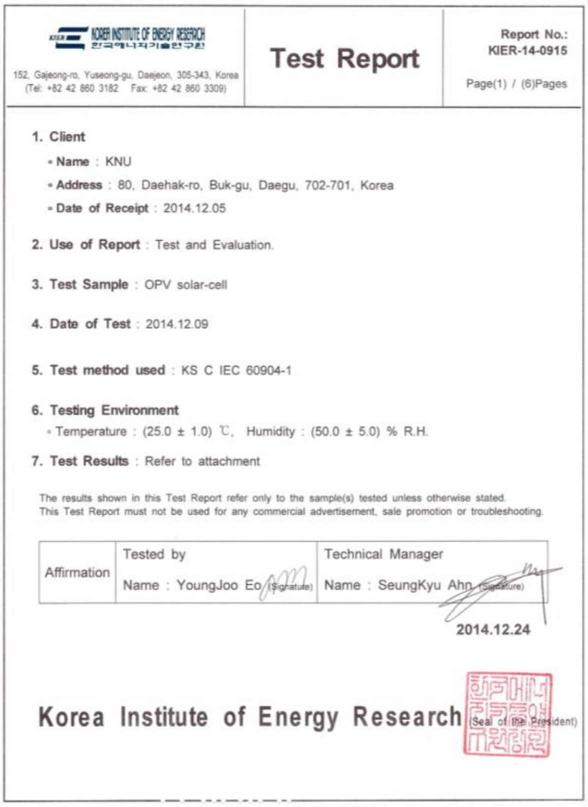

KIER KOREA INSTITUTE OF ENERGY RESEARCH 한국에너지기술연구원

152, Gajeong-ro, Yuseong-gu, Daejeon, 305-343, Korea
(Tel: +82 42 860 3182 Fax: +82 42 860 3309)

Test Report

Report No.: KIER-14-0915

Page(1) / (6)Pages

1. Client
   - Name : KNU
   - Address : 80, Daehak-ro, Buk-gu, Daegu, 702-701, Korea
   - Date of Receipt : 2014.12.05
2. Use of Report : Test and Evaluation.
3. Test Sample : OPV solar-cell
4. Date of Test : 2014.12.09
5. Test method used : KS C IEC 60904-1
6. Testing Environment
   - Temperature : (25.0 ± 1.0) ℃, Humidity : (50.0 ± 5.0) % R.H.
7. Test Results : Refer to attachment

The results shown in this Test Report refer only to the sample(s) tested unless otherwise stated.
This Test Report must not be used for any commercial advertisement, sale promotion or troubleshooting.

Affirmation	Tested by	Technical Manager
	Name : YoungJoo Eo (Signature)	Name : SeungKyu Ahn (Signature)

2014.12.24

Korea Institute of Energy Research (Seal of the President)

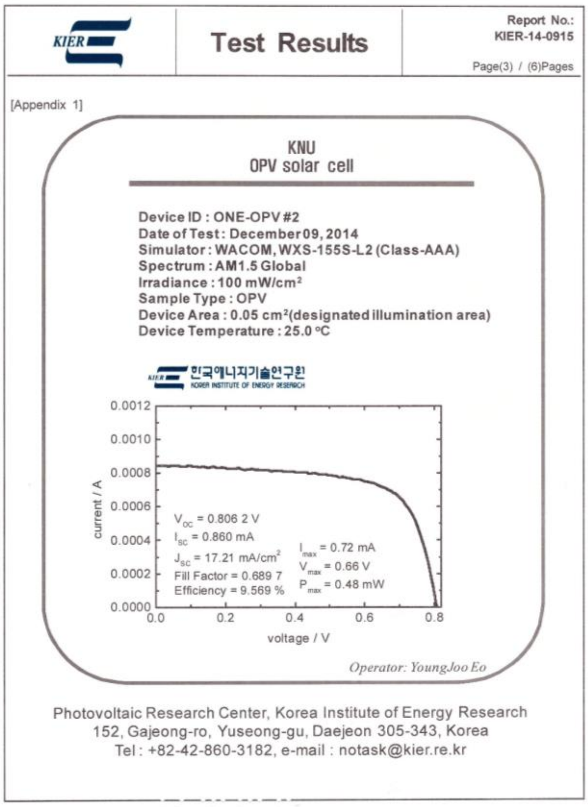

KIER

Test Results

Report No.: KIER-14-0915

Page(3) / (6)Pages

[Appendix 1]

KNU
OPV solar cell

Device ID : ONE-OPV #2
Date of Test : December 09, 2014
Simulator : WACOM, WXS-155S-L2 (Class-AAA)
Spectrum : AM1.5 Global
Irradiance : 100 mW/cm²
Sample Type : OPV
Device Area : 0.05 cm²(designated illumination area)
Device Temperature : 25.0 °C

Operator: YoungJoo Eo

Photovoltaic Research Center, Korea Institute of Energy Research
152, Gajeong-ro, Yuseong-gu, Daejeon 305-343, Korea
Tel : +82-42-860-3182, e-mail : notask@kier.re.kr

Supplementary Figure 1. Certificate of device performance. Copy of the certified results for the inverted PTB7-Th:$PC_{71}BM$ solar cells with the ZnO/PEOz(4) layer. The solar cell measurements were carried out by the National Solar Cell Accreditation Center of Korea Institute of Energy Research (KIER). Note that the device test was performed in air ambient condition so that the resulting device performance might be slightly lowered owing to the degradation by the attack of environmental moisture and oxygen. A black shadow mask was attached on the front glass side of the solar cell in order to avoid a waveguide effect by surrounding glass substrate that is not included in the active area.

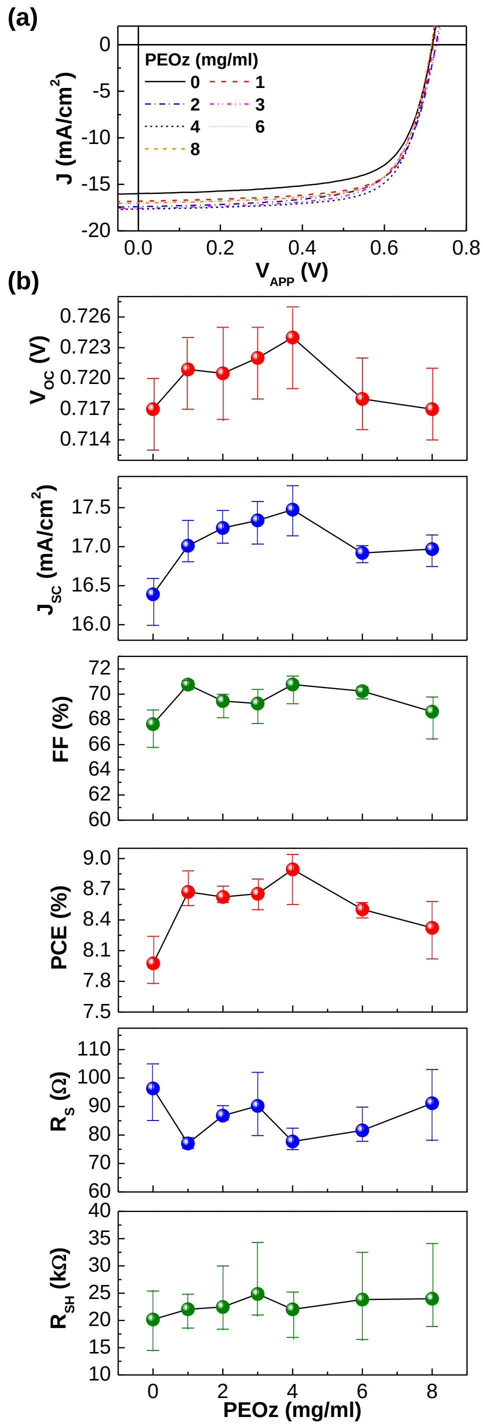

Supplementary Figure 2. Solar cell performances. (a) Light J-V curves of the inverted-type PTB7:$PC_{71}BM$ solar cells under one sun condition (air mass 1.5G, 100 mW/cm^2). (b) J_{SC}, V_{OC}, FF, PCE, R_S, and R_{SH} for the PTB7:$PC_{71}BM$ solar cells as a function of PEOz concentration. Note that the error bars were taken from more than 15 devices.

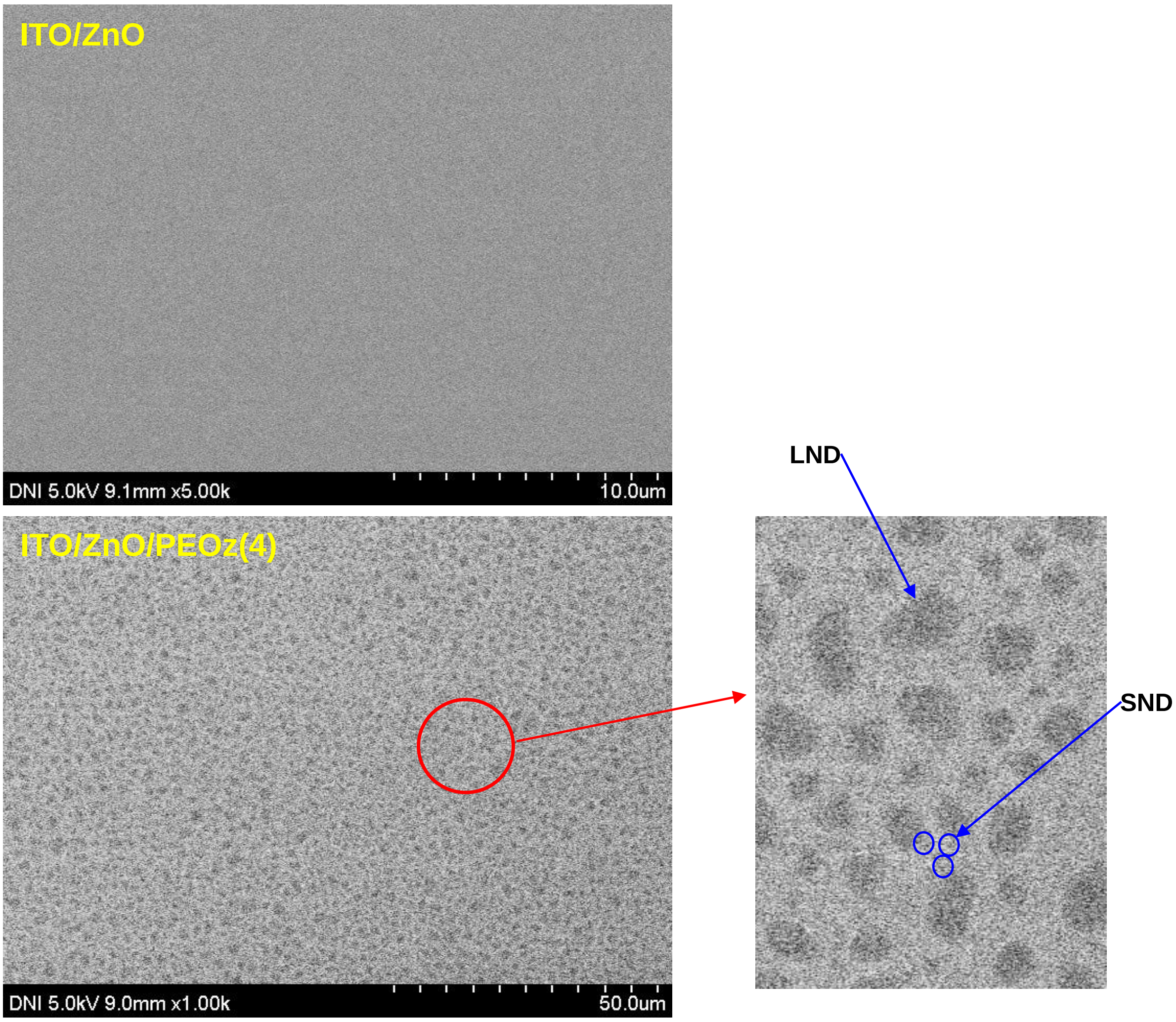

Supplementary Figure 3. Surface morphology. SEM images for the ZnO surface with and without the PEOz(4) layer: (a) ITO/ZnO (top), (b) ITO/ZnO/PEOz(4) layer (bottom). 'SND' and 'LND' denote 'small nano-dots' and 'large nano-dots in the enlarged image (right), respectively.

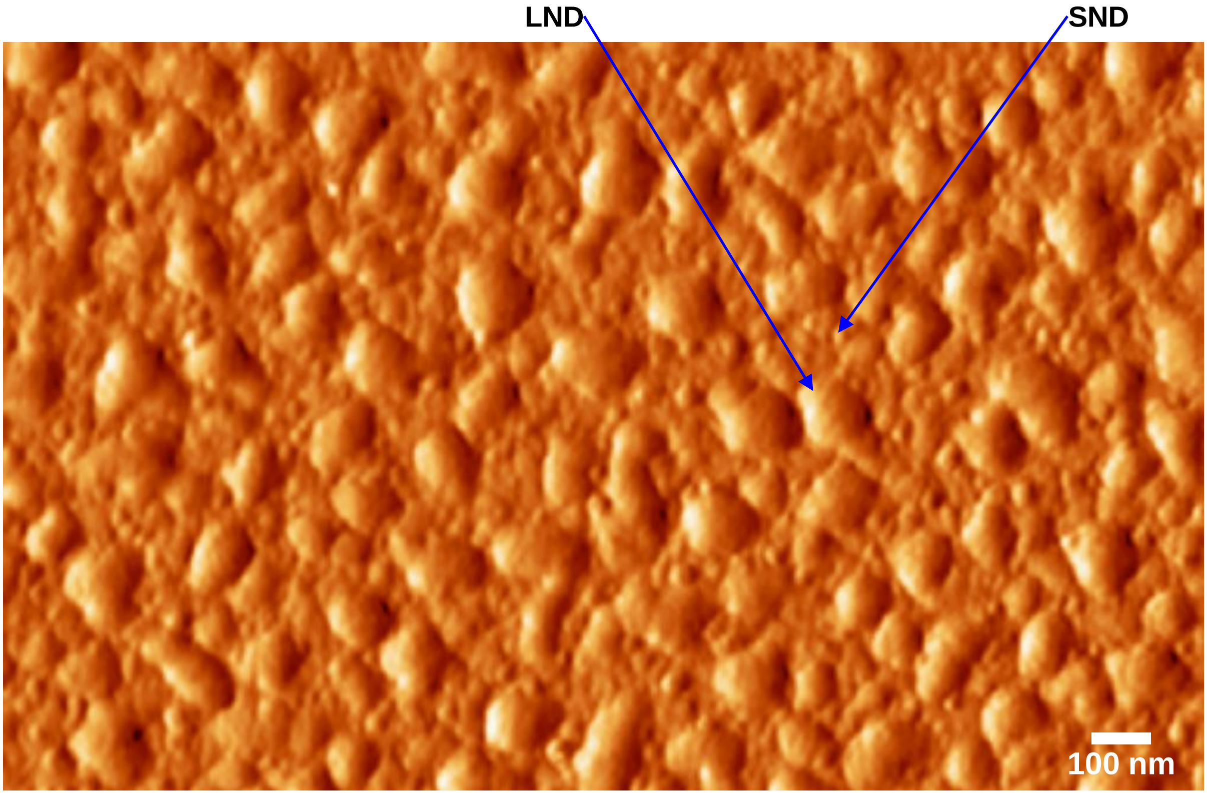

Supplementary Figure 4. Nano-dot structures. The enlarged phase-mode AFM image in Fig. 5(b) in order to clearly see the co-existence of both small nano-dots (SND) and large nano-dots (LND).

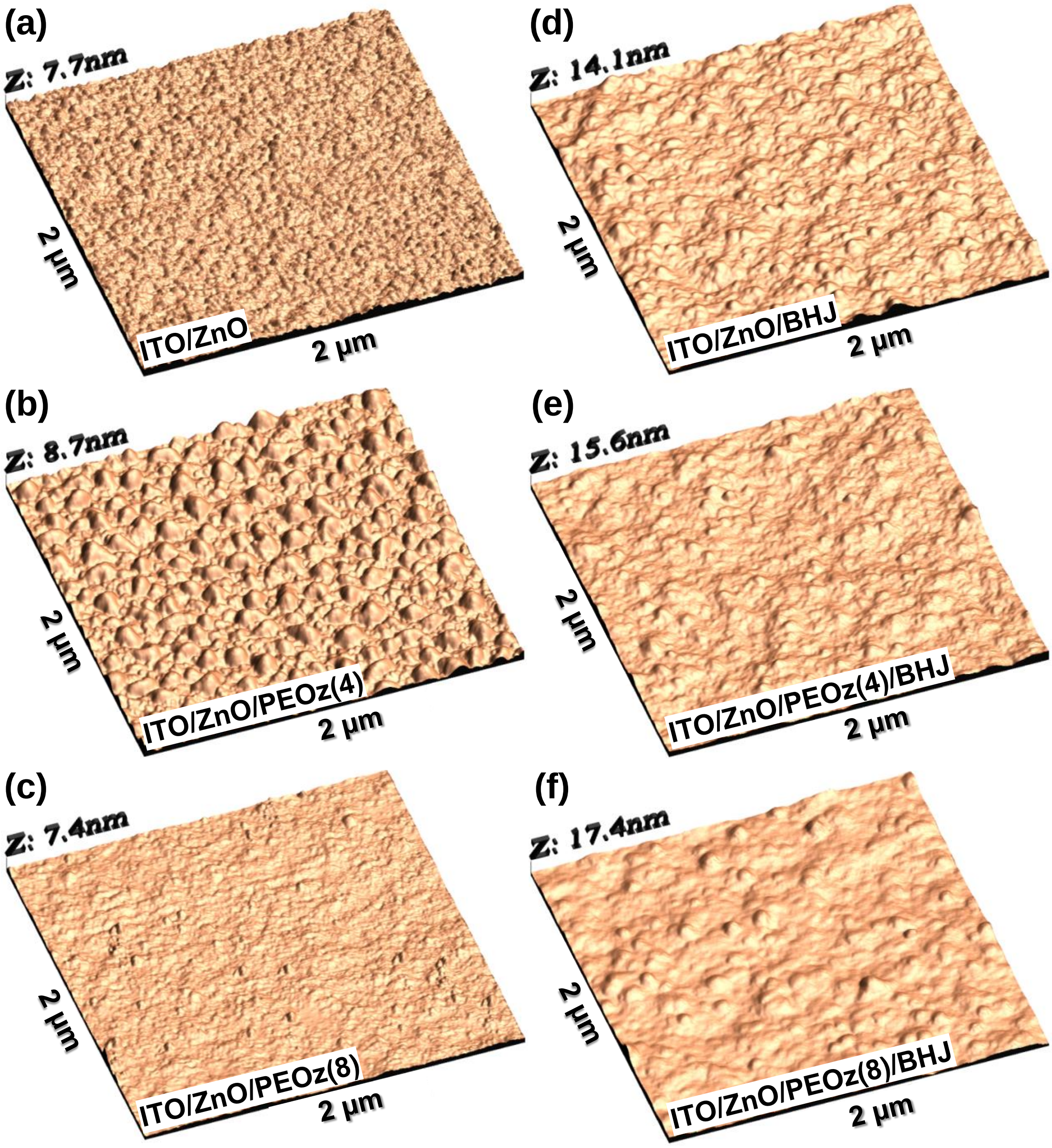

Supplementary Figure 5. Surface morphology. 3D height-mode of AFM images: (a) ITO/ZnO, (b) ITO/ZnO/PEOz(4), (c) ITO/ZnO/PEOz(8), (d) ITO/ZnO/BHJ, (e) ITO/ZnO/PEOz(4)/BHJ, (f) ITO/ZnO/PEOz(8)/BHJ. The 'BHJ' layer represents 'PTB7-Th:$PC_{71}BM$' layer.

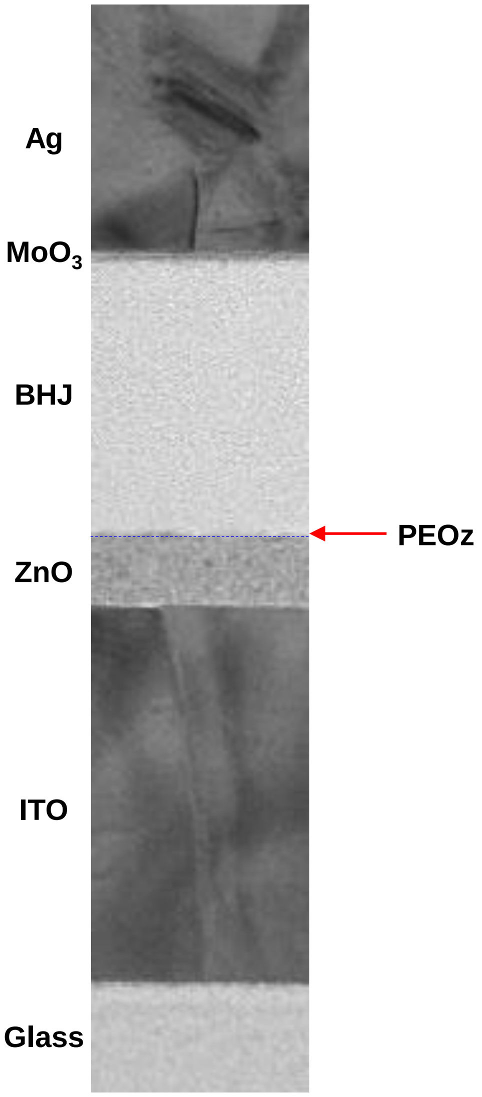

Supplementary Figure 6. Cross-section structure of device. HRTEM image for the cross-section part of the PTB7-Th:PC_{71}BM solar cell with the PEOz(4) nanolayer. Note that the device cross-section sample was obtained by using the FIB system. The shape of the PEOz(4) in this image is unclear but vaguely observed because of the very thin state.

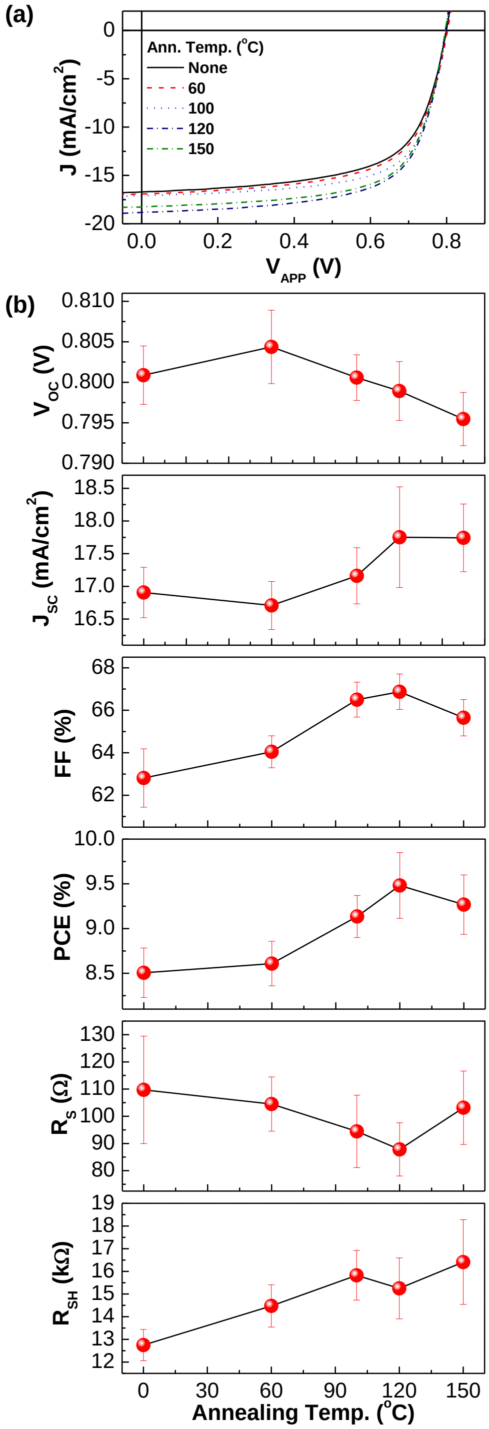

Supplementary Figure 7. **Solar cell performances.** (a) Light J-V curves of the inverted-type PTB7-Th:PC_{71}BM solar cells with the PEOz(4) layer according to the thermal annealing temperature under one sun condition (air mass 1.5G, 100 mW/cm^2). (b) J_{SC}, V_{OC}, FF, PCE, R_S, and R_{SH} for the inverted-type PTB7-Th:PC_{71}BM solar cells with the PEOz(4) layer as a function of the thermal annealing temperature. Note that the error bars were taken from more than 10 devices.

Supplementary Table 1. Summary of recent high efficiency results (PCE > 9.5 %) reported for single-stack polymer:fullerene solar cells.

No	Author (Affiliation)	Active Materials	Device Structure	Key Points	Best PCE (%)	Year	Ref
1	Y. Liu (Hong Kong Uni.)	PffBT4T-2OD:$PC_{71}BM$	Inverted	Temperature-dependent aggregation	10.8	2014	S1
2	S.-H. Liao (National Tsing-Hua Uni.)	PTB7-Th:$PC_{71}BM$	Inverted	InZnO-$BisC_{60}$ electron collection layer	10.3	2014	S2
3	C. Liu (Uni. Akron)	PTB7-DT:$PC_{71}BM$	Normal	Introduction of 5-alkylthiophene-2-yl side chains	10.1	2015	S3
4	Z. He (South China Uni. Tech.)	PTB7-Th:$PC_{71}BM$	Inverted	$PC_{71}BM$ weight fractions	10.6	2015	S4
5	This Work	PTB7-Th:$PC_{71}BM$	Inverted Structure	PEOz nano-dot interfacial layer	10.7	2015	-

Supplementary References

1. Y. Liu, J. Zhao, Z. Li, C. Mu, W. Ma, H. Hu, K. Jiang, H. Lin, H. Ade, and H. Yan, *Nat. Commun.*, **2014**, *5*, 5293, DOI: 10.1038/ncomms6293.

2. S.-H. Liao, H.-J. Jhuo, P.-N. Yeh, Y.-S. Cheng, Y.-L. Li, Y.-H. Lee, S. Sharma, S.-A. Chen, *Sci. Rep.*, **2014**, *4*, 6813, DOI: 10.1038/srep06813.

3. C. Liu, C. Yi, K. Wang, Y. Yang, R. S. Bhatta, M. Tsige, S. Xiao, and X. Gong, *ACS Appl. Mater. Interfaces* **2015**, *7*, 4928-4935.

4. Z. He, B. Xiao, F. Liu, H. Wu, Y. Yang, S. Xiao, C. Wang, T. P. Russell, and Y. Cao, *Nat. Photon.*, **2015**, *9*, 174.
